# Cluster Individuals Based on Phenotype and Determine the Risk for Atrial Fibrillation in the PREVEND and Framingham Heart Study Populations

**DOI:** 10.1371/journal.pone.0165828

**Published:** 2016-11-10

**Authors:** Michiel Rienstra, Bastiaan Geelhoed, Xiaoyan Yin, Joylene E. Siland, Rob A. Vermond, Bart A. Mulder, Pim Van Der Harst, Hans L. Hillege, Emelia J. Benjamin, Isabelle C. Van Gelder

**Affiliations:** 1 Department of Cardiology, University of Groningen, University Medical Center Groningen, Groningen, The Netherlands; 2 National Heart Lung and Blood Institute's and Boston University's Framingham Heart Study, Framingham, Massachusetts, United States of America; 3 Section of Cardiovascular Medicine, Department of Medicine, Boston University School of Medicine, Boston, Massachusetts, United States of America; 4 Section of Preventive Medicine, Department of Medicine, Boston University School of Medicine, Boston, Massachusetts, United States of America; 5 Department of Epidemiology, Boston University School of Public Health, Boston, Massachusetts, United States of America; Universita degli Studi di Napoli Federico II, ITALY

## Abstract

**Background:**

Risk prediction of atrial fibrillation (AF) is of importance to improve the early diagnosis and treatment of AF. Latent class analysis takes into account the possible existence of classes of individuals each with shared risk factors, and maybe a better method of incorporating the phenotypic heterogeneity underlying AF.

**Methods and findings:**

Two prospective community-based cohort studies from Netherlands and United States were used. Prevention of Renal and Vascular End-stage Disease (PREVEND) study, started in 1997, and the Framingham Heart Study (FHS) Offspring cohort started in 1971, both with 10-years follow-up. The main objective was to determine the risk of AF using a latent class analysis, and compare the discrimination and reclassification performance with traditional regression analysis. Mean age in PREVEND was 49±13 years, 49.8% were men. During follow-up, 250(3%) individuals developed AF. We built a latent class model based on 18 risk factors. A model with 7 distinct classes (ranging from 341 to 1517 individuals) gave the optimum tradeoff between a high statistical model-likelihood and a low number of model parameters. All classes had a specific profile. The incidence of AF varied; class 1 0.0%, class 2 0.3%, class 3 7.5%, class 4 0.2%, class 5 1.3%, class 6 4.2%, class 7 21.7% (p<0.001). The discrimination (C-statistic 0.830 vs. 0.842, delta-C -0.013, p = 0.22) and reclassification (IDI -0.028, p<0.001, NRI -0.090, p = 0.049, and category-less-NRI -0.049, p = 0.495) performance of both models was comparable. The results were successfully replicated in a sample of the FHS study (n = 3162; mean age 58±9 years, 46.3% men).

**Conclusions:**

Latent class analysis to build an AF risk model is feasible. Despite the heterogeneity in number and severity of risk factors between individuals at risk for AF, latent class analysis produces distinguishable groups.

## Introduction

Atrial fibrillation (AF) is the most common sustained cardiac arrhythmia and is not a benign condition.[1–5] Despite the fact that in the last decades many risk factors for AF, such as advancing age, hypertension, obesity, diabetes, and cardiovascular diseases, such as heart failure, valve disease, and myocardial infarction, have been identified, the development of AF and its complications remains highly variable.[5–8] Some AF patients have multiple risk factors, where others have none; others have multiple risk factors but never develop AF. Traditional risk-factor-based AF prediction models are far from ideal and do not account for the wide biological heterogeneity underlying AF risk.[9,10] Adequate risk assessment is of utmost importance to improve the utilization of diagnostic tools to detect AF in those at risk for AF, and to apply therapeutic strategies to prevent AF and its related morbidity and mortality.[11,12]

Latent class analysis is a statistical method that can be used for risk prediction, taking into account the possible existence of classes of individuals each with a different distribution of cardiovascular risk factors and diseases. Latent class analysis has been successfully applied in complex diseases as asthma, attention deficit hyperactivity disorder, and amyotrophic lateral sclerosis, and recently in heart failure.[13–17] Latent class analysis is a probabilistic clustering approach assuming that associations between directly measured variables are caused by a latent parameter, which itself is not directly measurable, but can be inferred from the directly measured variables.[18]

We aim to determine the risk of AF in individuals of the community-based PREVEND study (Netherlands), using a latent class analysis, and compare the discrimination and reclassification performance with traditional Cox regression analysis-based AF risk prediction, and validate the risk model based on latent class analysis in the Framingham Heart Study (FHS) (United States).

## Methods

### Population

Our study was performed using data from the PREVEND study, founded in 1997 in Groningen, The Netherlands. A detailed description of PREVEND has been previously reported.[5] In total, 8,592 individuals were included and followed at three-year intervals. For present analysis, we excluded 248 individuals without any electrocardiogram (ECG), as well as 79 individuals with prevalent AF. The study was approved by the institutional Medical Ethics Committee and conducted in accordance with the Declaration of Helsinki. All individuals provided written informed consent.

### Definitions

Incident AF ascertainment has been described in detail previously.[5] Atrial fibrillation was diagnosed on ECGs made at study visits, outpatient visits and hospitalizations. Blood pressures were calculated as the mean of the last two measurements, using an automatic Dinamap XL Model 9300 series device. Body mass index (BMI) was calculated as the ratio of weight to height squared (kg/m^2^), and obesity was defined as a BMI >30.0 kg/m^2^. Type 2 diabetes was defined as a fasting plasma glucose >7.0 mmol/L (126 mg/dL), a nonfasting plasma glucose >11.1 mmol/L, or use of anti-diabetic medication. Smoking was defined as current nicotine use or quit smoking within the previous five years. Hypercholesterolemia was defined as total serum cholesterol >6.5 mmol/L (251 mg/dL) or a serum cholesterol ≥5.0 mmol/L (193/mg/dL) if a history of myocardial infarction was present or use of lipid-lowering medication. Previous myocardial infarction or stroke was defined as individual-reported hospitalization for at least three days for one of both conditions. A committee of heart failure experts adjudicated all individuals with heart failure at inclusion according to previously published criteria. Peripheral artery disease was defined as an ankle-brachial index *<*0.9. The glomerular filtration rate (eGFR) was estimated using the simplified modification of diet in renal disease formula. Urinary albumin excretion was calculated as the average value from two consecutive 24-hour urine collections.

### Follow up

The follow-up duration was calculated as the time between the baseline-screening visit and incident AF, death, or the last contact date to a maximum of 10 years.

### Validation sample

We used participants of the FHS Offspring cohort, who attended the 6^th^ examination cycle (1995–1998; n = 3,532) as a validation cohort. Individuals with prevalent AF (n = 94), missing GFR (n = 66), 75 years or older (n = 184), or missing any covariate (n = 26) were excluded. A detailed description of FHS has been previously published.[19] Definitions of covariates and follow-up were the same as in the PREVEND study, with the following differences. Individuals were defined to have AF if AF or atrial flutter was confirmed by a FHS cardiologist on review of ECGs. Smoking was defined as current nicotine use if the participant smoked cigarettes regularly within the previous one year. Hypercholesterolemia was defined as total serum cholesterol >6.5 mmol/L (251 mg/dL) or use of lipid-lowering medication. Previous myocardial infarction or stroke were diagnosed by review of hospital records and physician reports, and adjudicated by three FHS investigators. Peripheral artery disease was defined as experiencing any of the following conditions: percutaneous transluminal coronary angioplasty, carotid artery surgery, aorta surgery and femoral of lower extremity surgery.

### Statistical analysis

Latent class analysis was performed using the poLCA function in the R statistical package.[20] Since poLCA requires polytomous variables, we converted all continuous covariates into categorical variables based on tertiles or quantiles. Individuals’ characteristics were presented as counts with percentages, mean±SD, or median (interquartile range). Latent class analysis was performed based on pre-specified cardiovascular risk factors and diseases. We excluded 1093 individuals from the latent class model fitting because of missing values in any of the class determiningvariables. Because in PREVEND there was an overrepresentation of individuals with urinary albumin excretion ≥10mg/L at study start, we added urinary albumin excretion ≥10mg/L as a class-defining variable in all the latent class analyses and as a covariate in all the Cox proportional hazards regressions. According to the method by Lanza et al.,[21] we included incident AF (study outcome) as covariate (**Fig A in S1 File**) to perform the cluster analysis. When applying the latent class model, the predicted risk of AF is the conditional probability of AF given the covariates ((incident AF covariate was excluded). This conditional probability does not depend on actual AF status of an individual (see the **Supplementary Methods in S1 File**) A prerequisite of latent class clustering analysis is the local independence of the included variables within each latent class. Therefore, we excluded a variable (except for AF, age, sex and urinary albumin excretion ≥10 mg/L which were included in all analyses) when the correlation coefficient was >0.4 with another variable, in order to avoid too strong a correlation between variables. The order of exclusion variables was based on the total number of strong correlations. A priori, the number and size of the latent classes was unknown. For reasons of generalizability and practicability, we aimed for the smallest number of class-defining variables in the latent class model. The construction of latent classes is achieved by maximizing the log-likelihood. For optimization, <20,000 iterations of the poLCA algorithm (applies both the Expectation-Maximization and the Newton-Raphson algorithms) were sufficient to reach convergence to a maximum. To reduce the influence of local maximums during the poLCA algorithm, the algorithm was initiated ten times with different random initialization matrices of the latent probabilities. The model fit with the highest likelihood was selected. To estimate the optimum number of classes, we compared the fit of models with increasing numbers of classes. The number of classes for which the Bayesian information criterion (BIC) had a minimum value was taken as the optimum number of classes. Additional model fit statistics were calculated, including the Akaike information criterion, normalized Chi-squared (Pearson Chi-square for model fit divided by the number of residual degrees of freedom), expected size of the smallest class, log-likelihood, Madansky’s measure for local independence,[22] and root mean square error. We ran an internal validation of the optimum latent class clustering analysis fit by performing parametric bootstrapping to estimate the p-values of the normalized Chi-squared, the log-likelihood, Madansky’s measure for local independence, and root mean square error. The risk of AF was estimated using two methods. First, latent class analysis was used to estimate the risk of AF of each class. Second, each individual (all 8265 individuals) was, based on maximum posterior probability, assigned to its most likely class, and subsequently Kaplan-Meier analysis was performed to calculate the cumulative event proportions for each class, and the log-rank test was used to compare classes. More detailed explanation of log-likelihood, Bayesian information criterion, Akaike information criterion, root mean square error, Madansky measure, and posterior probability classification in the Supplementary Methods.

As comparative model, we built a traditional risk-factor-based model using Cox proportional hazards regression analysis, and included the AF risk factors in the CHARGE-AF risk prediction model.[10] To investigate the discrimination and reclassification performance of the latent-class model, we compared with the traditional risk-factor-based model. We examined the C-statistic for binary data, reclassification and discrimination of predicted AF risk with integrated discrimination, and net reclassification improvement indexes.[23] We used risk thresholds of less than 5%, 5% to 10%, and greater than 10% for ten years of follow-up for the net reclassification improvement index. We performed an independent validation analysis in 3162 persons enrolled in the FHS. The latent class model built using the PREVEND data was applied to the FHS sample. Performance, reclassification, and discrimination indices were calculated. All analyses were performed using R package (version 3.03), and a two-sided p-value <0.05 was considered statistically significant. We used the TRIPOD criteria for transparent reporting.[24]

## Results

### Sample characteristics and incidence of AF

Mean age was 48.9 years and 49.8% were men. The sample characteristics are described in **Table 1**. Mean follow-up was 9.2±2.1 years (76,094 person-years). Of the 8,265 individuals, 250 (76 women, 174 men) individuals (3.0%) developed AF.

**Table 1:** Cardiovascular risk factors and diseases in the PREVEND and Framingham Heart Study samples.

	PREVEND Study (n = 8,265)	Framingham Heart Study(n = 3162)
Age (years)		
≤35	1458(17.6%)	17(0.5%)
36–43 years	1717(20.8%)	165(5.2%)
44–50 years	1611(19.5%)	552(17.5%)
51–61 years	1740(21.1%)	1301(41.1%)
≥62 years	1739(21.0%)	1127(35.6%)
Men	4120(49.8%)	1463(46.3%)
European ancestry	7844(94.9%)	3162(100.0%)
Height (cm)	173(166–180)	167(161–175)
Weight (kg)	77(68–87)	77(66–89)
BMI		
≤22 kg/m^2^	1634(20.0%)	406(12.8%)
23–24 kg/m^2^	1635(20.0%)	446(14.1%)
25–26 kg/m^2^	1636(20.0%)	572(18.1%)
27–29 kg/m^2^	1635(20.0%)	679(21.5%)
≥30 kg/m^2^	1636(20.0%)	1059(33.5%)
Diastolic blood pressure	74±10	76±9
Systolic blood pressure	129±20	128±18
Heart rate		
≤63 bpm	2417(29.4%)	1639(51.8%)
64–72 bpm	2950(35.7%)	970(30.7%)
≥73 bpm	2854(34.5%)	553(17.5%)
Antihypertensive therapy	1098(13.3%)	839(26.5%)
Previous myocardial infarction	251(3.0%)	86(2.7%)
Heart failure	18(0.2%)	17(0.5%)
Diabetes mellitus	310(3.8%)	286(9.0%)
Previous stroke	81(1.0%)	52(1.6%)
Peripheral artery disease	291(3.5%)	84(2.7%)
Smoking	3670(44.4%)	507(16.0%)
Hypercholesterolemia	1235(14.9%)	715(22.6%)
Alcohol use	1054(12.8%)	73(2.3%)
PR interval duration (ms)		
≤149	2679(33.2%)	751(23.7%)
150–166	2447(29.6%)	1204(38.1%)
≥167	2937(35.5%)	1207(38.2%)
Glomerular filtration rate		
≤74 ml/min	2734(33.3%)	831(26.3%)
75–86 ml/min	2731(33.3%)	793(25.1%)
≥87 ml/min	2741(33.4%)	1538(48.6%)
Urinary albumin excretion ≥ 10 mg/L	5759(69.7%)	-

Data are expressed as numbers (%), mean±SD, or median [25^th^– 75^th^ percentile].

### Latent class model of cardiovascular risk factors and diseases

**Fig 1:**
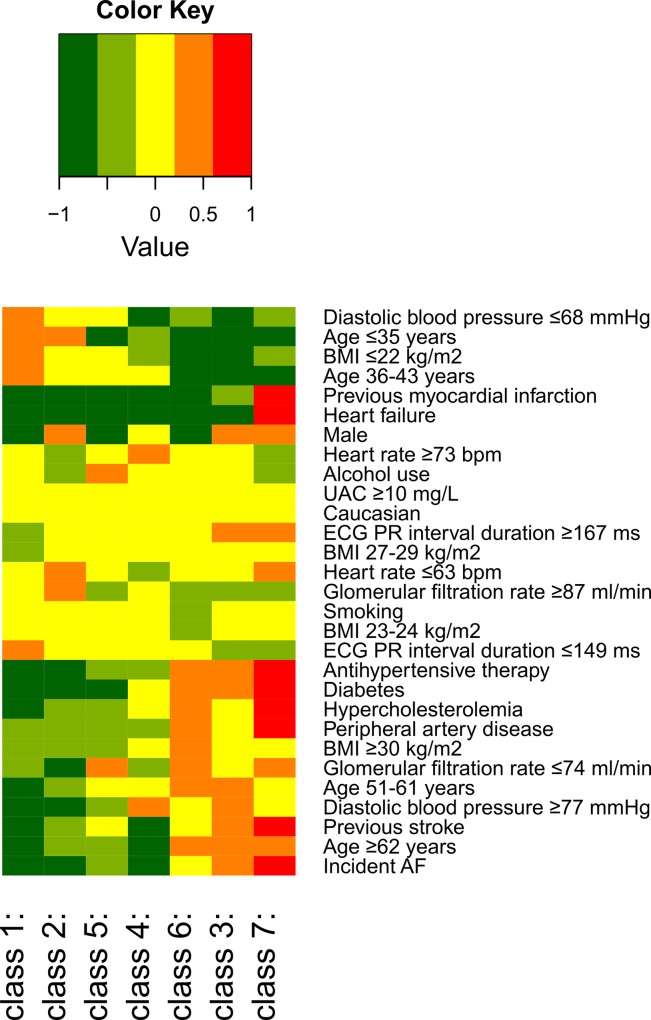
Heat coloring map of individual characteristics within each latent class based on the latent- class model including cardiovascular risk factors, diseases, and AF. The probability ratio R (probability of having the characteristic within a class divided by the probability of having the characteristic within the entire population) is represented by colors. Dark green = strongly reduced probability ratio (R< 10^−0.6^), light green = reduced probability ratio (R = 10−0.6–10^−0.2^), yellow = unchanged or weakly reduced/increased probability ratio (R = 10−0.2–10^0.2^), orange = increased probability ratio (R = 100.2–10^0.6^), and red = strongly increased probability ratio (R>10^0.6^).

**Fig 2:**
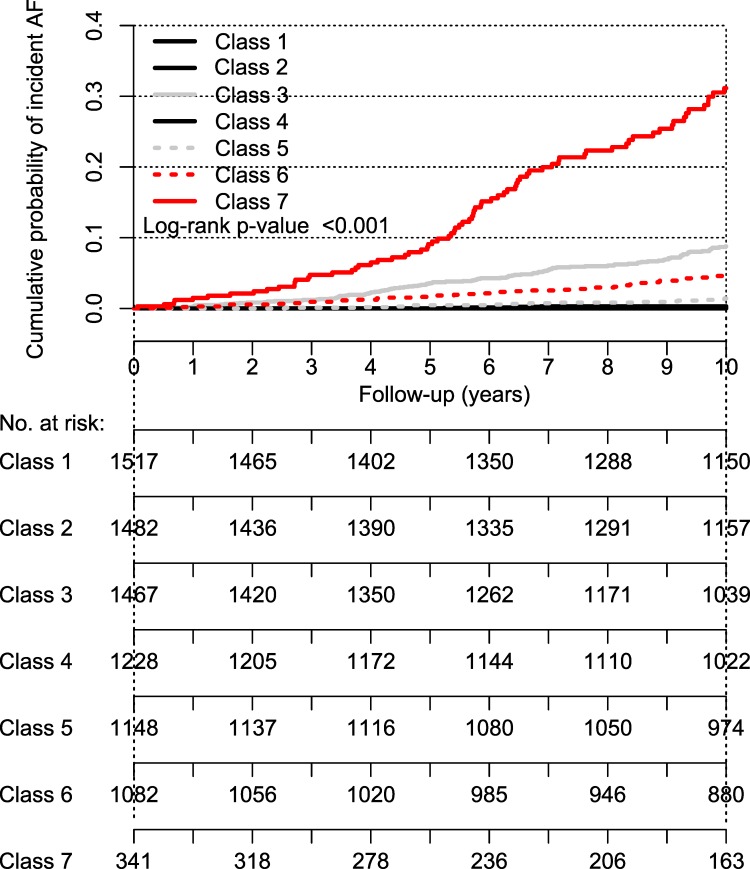
Cumulative risk of AF according to the 7 clusters.

We built a latent class model based on the following cardiovascular risk factors, and diseases; age, men, European ancestry, BMI, diastolic blood pressure, heart rate, antihypertensive therapy, previous myocardial infarction, heart failure, diabetes, prior stroke, peripheral artery disease, smoking, alcohol use, hypercholesterolemia, PR-interval duration, glomerular filtration rate, urinary albumin excretion, and incident AF. The covariates hypertension and systolic blood pressure were excluded because of too strong correlations with one or more of the included characteristics. The model with 7 distinct classes gave the best fit (**Table 2**), based on the lowest BIC value; an optimum tradeoff between a high statistical likelihood of the model and a low number of parameters needed for specifying the model. Parametric bootstrapping estimating the p-values of the normalized Chi-squared (805.7; p = 0.38), the log-likelihood -78,244; p = 0.19), Madansky’s measure for local independence (34.980×10^6^; p = 0.38), and root mean square error statistic (0.021; p = 0.30), was performed as internal validation of the optimum number of classes (n = 7), and were all non-significant, implying good model fit. All the classes were distinguishable and had a specific pattern of risk factors and diseases. In **Fig 1** and **Table A in S1 File** the characteristics of each class were shown. Class 1 was the largest with 1517 young women without cardiovascular risk factors, whereas class 7 was the smallest with 341 predominantly elderly with high prevalence of risk factors and diseases. Class 2 to 6 all included >1000 individuals. Class 2 and 3 consisted of men, with in class 3 more older individuals with more risk factors like higher blood pressure and diabetes. Class 4 included relatively young individuals, a large proportion over-weighted and with higher blood pressure, but almost no diabetes. Class 5 consisted of middle-aged individuals with a large proportion of alcohol users. Class 6 consisted of women with relatively high prevalence of risk factors, though not as much as in class 7. The incidence of AF varied in each class, and was 0.0% for class 1, 0.3% for class 2, 7.5% for class 3, 0.2% for class 4, 1.3% for class 5, 4.2% for class 6, and 21.7% for class 7. The cumulative incidence of AF according to the classes is depicted in **Fig 2**.

**Table 2:** Fit statistics of latent class clustering of cardiovascular risk factors and diseases (primary analysis with AF as class-determining variable).

Nr. of classes	BIC	AIC	Normalized Chi-squared	Expected size of smallest class	Log-likelihood	Madansky measure/10^6^	RMSE
1	166372.0	166172.6	73078.8	7172	-83057.3	39.571	0.000
2	160556.8	160151.0	6413.0	3001	-80016.5	35.909	0.006
3	159466.2	158854.1	5633.3	1956	-79338.0	34.555	0.005
4	158823.8	158005.3	2399.8	1371	-78883.6	34.633	0.012
5	158428.6	157403.7	1668.7	995	-78552.9	34.403	0.014
6	158369.9	157138.8	855.2	499	-78390.4	34.307	0.019
7	158342.8	156905.3	805.7	281	-78243.7	34.980	0.021
8	158397.4	156753.6	826.3	320	-78137.8	35.280	0.020
9	158473.2	156623.0	850.0	204	-78042.5	35.287	0.021
10	158567.6	156511.1	763.9	196	-77956.5	35.244	0.022

Abbreviations: AIC = Akaike information criterion; BIC = Bayesian information criterion; RMSE = root-mean-square-error.

### Utility of latent class-based modeling for AF risk prediction

In the primary analysis, the discrimination of the latent-class-based model was compared to the traditional risk-factor-based model with previously published risk factors of AF (**Table 3**). The C-statistic of the traditional risk-factor-based model was 0.842 (95% confidence interval 0.820–0.864). The C-statistic of the clustering model the was 0.830 (95% confidence interval 0.806–0.853), and comparable to the traditional risk-factor-based model (delta C-statistic p = 0.22). The prognosticated event rate for the latent-class-based model was 2.8%, for the traditional risk-factor-based model 2.9%, both close to the observed event rate of 3%. The traditional risk-factor-based model performed better than the cluster-based model with respect to the integrated discrimination improvement index and net reclassification index. However, with respect to the category-less net reclassification improvement index, there was no statistically significant difference in performance between the cluster-based model and the traditional risk-factor-based model. (**Table 3**).

**Table 3:** Discrimination and reclassification performance of latent class clustering models and comparison with traditional risk-factor-based AF prediction model*.

	C-statistic		Integrated discrimination improvement index		Net reclassification improvement index		Category-less net reclassification improvement index	
	Statistic (95% CI)	P-value**	Statistic (95% CI)	P-value**	Statistic (95% CI)	P-value**	Statistic (95% CI)	P-value**
**PREVEND Study**								
Traditional risk-factor-based AF model	0.842(0.820 to 0.864)	-	-	-	-	-	-	-
Cluster-based model	0.830(0.806 to 0.853)	0.22	-0.028(-0.043 to -0.013)	<0.001	-0.090(-0.180 to 0.000)	0.049	-0.049(-0.189 to 0.092)	0.495
**Framingham Heart Study**								
Traditional risk-factor-based AF model	0.725(0.690 to 0.760)	-	-	-	-	-	-	-
Cluster-based model	0.704(0.666 to 0.742)	0.13	-0.018(-0.031 to -0.005)	0.007	0.007(-0.084 to 0.098)	0.877	-0.153(-0.290 to -0.016)	0.029

*Traditional risk-factor-based AF prediction model includes age, sex, height, weight, antihypertensive drug use, systolic blood pressure, diastolic blood pressure, smoking, diabetes, heart failure, previous myocardial infarction, and race.[10]

**Compared with the traditional risk-factor-based AF model. Abbreviations: AF = atrial fibrillation; CI = confidence interval.

### Validation of clustering model

As validation, we applied the latent-class-model and the traditional risk factor-based model to the Framingham Heart Study cohort (n = 3,162). The sample characteristics are described in **Table 1**. Two-hundred-twelve individuals developed incident AF (6.7%). The C-statistic of the traditional risk-factor-based model was 0.725 (95% confidence interval 0.690–0.760). The C-statistic of the latent-class model was 0.704 (95% confidence interval 0.666–0.742). The difference between these two C-statistics was not statistically significant (delta C statistic p = 0.13). The traditional risk factor-based model performed better than the cluster-based model with respect to the integrated discrimination improvement index, and category-less net reclassification improvement index, but not regarding the net reclassification index (**Table 3**). The latent probabilities of the latent class model and the regression coefficients of the Cox model are given in **Table B in S1 File** and **Table C in S1 File**. The cumulative hazard of the traditional risk factor-based model is given in **Fig B in S1 File**.

## Discussion

We demonstrated, as proof-of-principle, that applying a probabilistic latent class clustering approach can identify classes of individuals with similar cardiovascular risk factors and diseases, and the classes themselves were distinguishable from another. The risk of AF was different for each class. The performance of the latent-class-based models was comparable to a traditional risk-factor-based model predicting risk of future AF, and was successfully validated in an independent cohort.

To our knowledge, latent class clustering analysis has not been applied to predict the risk of AF before. What are the potential advantages of this method compared to traditional risk-factor based risk prediction? First, the major difference between latent-class-based models and traditional risk-factor-based models is the fact that latent class clustering centers on individuals and not on risk factors. Within each cluster, individuals have a similar clinical phenotype, and may also share the underlying pathophysiology. The insights into the pathophysiology underling AF risk suggested by latent classes may have important implications for both clinicians and researchers. For clinicians, identifying individuals at high risk for AF, may help to develop diagnostic strategies to detect AF, and apply treatment strategies targeting the involved predominant pathway, may increase treatment benefit, and reduce adverse effects. We found 7 distinct classes. The extremes; the class including young women without cardiovascular risk factors or diseases, and on the other end; the class with individuals with multiple cardiovascular risk factors and diseases, are completely in line with what clinicians do by instinct when encountering patients. However, the intermediate groups are more complicated to distinguish from one other, without using methods such as cluster analysis. In addition to the two extremes, cluster analysis revealed another 5 clusters, with clearly different characteristics and AF-risks. Since latent class clustering centers on individuals and not on risk factors it may be easier to translate results to the individual patient, and tailored therapy may be within reach, although more research is needed.[25]

Also, dealing with large amounts of data as collected from individuals is an advantage of latent clustering analysis. Currently, the risk of AF or its complications for an particular individual is calculated using easy-to-remember tools, disregarding the detailed information collected.[26] In the future, tools based on latent clustering analyses may facilitate the risk prediction by incorporating detailed phenotypic information.[26]

For researchers, identifying individuals with same pathophysiology may help to further study the predominant pathway in these subsets of individuals, and study in more detail the role of circulating biomarkers, and genetic susceptibility underlying AF. As we demonstrate here, the method can be used to determine the risk of complex diseases such as AF. The common denominator of complex diseases is the biological heterogeneity, and wide variability in the clinical presentation of those at risk for disease. Determining more homogeneous phenotypes with differential risk of AF may help to improve the understanding the susceptibility of complex disease, like AF.

Our study has substantive strengths, however also limitations. We developed and replicated our model in two longitudinally followed community-based cohorts with routine ascertainment of risk factors, cardiovascular disease, and AF. Since the aim of present study was to apply for the first time the latent class clustering methodology in AF, we decided to use an open-source latent class clustering program (PoLCA), to ensure that methods can be applied by others. An disadvantage of PoLCA is that it can only deal with dichotomized data, which may have led to underestimation of the performance of the latent class clustering model. However, dichotomizing has the advantage that no assumptions regarding normality of data are needed. We restricted our analysis to the traditional AF risk factors, and did not account for others, such as alcohol or physical activity. This may have led to an further underestimation of the performance of the latent class clustering model. Other limitations are mainly due to the observational design of the used community-based cohorts. Both studies comprised of white, largely middle-aged adults, so results cannot be directly generalized to other races/ethnicities, individuals outside the ages studied, or to the clinical context. We also acknowledge that AF not infrequently is clinically unrecognized, contributing to misclassification of the outcome. In addition, we did not distinguish between AF and atrial flutter and patterns of AF, which may have different latent class structures.

## Conclusion

Latent class clustering based AF risk prediction may help to unravel a distinct or predominant pathophysiological mechanism underlying individuals with shared cardiovascular risk factors. Use of latent class clustering to build a novel AF risk classification model is feasible.

## Supporting Information

S1 File Supplementary methods. **Table A.** PREVEND: Characteristics in the groups when each case is assigned to a group based on highest posterior probability of the latent class clustering analysis based on cardiovascular risk factors and diseases, including incident AF (primary analysis). **Table B.** PREVEND: The latent probabilities of the latent class model. **Table C.** Multivariable-adjusted Cox proportional hazards regression coefficients for 10-year risk of AF. **Fig A: Graphical representation of the latent class model with distal outcome.** C refers to the latent class variable. The class-defining variables of C are age (shown in the Fig), men (shown in the Fig), European ancestry, body mass index, diastolic blood pressure, heart rate, antihypertensive treatment, Previous myocardial infarction, heart failure, diabetes, previous stroke, peripheral artery disease, smoking, alcohol use, hypercholesterolemia, ECG PR interval duration, eGFR-creatinine-based <60, and UAC ≥ 10 mg/L (shown in the Fig). The outcome is incident AF. **Fig B: Underlying cumulative hazard function of the traditional risk factor-based model.** The PREVEND population was used to estimate the underlying cumulative hazard function of the traditional risk factor-based model. The solid line is the underlying cumulative hazard function of the traditional risk factor-based model, the dashed lines represent the 95% confidence interval.
(DOCX)
