# Supplementary material for: Cluster Individuals Based on Phenotype and Determine the Risk for Atrial Fibrillation in the PREVEND and Framingham Heart Study Populations
Source: PLoS One. 2016 Nov 10;11(11):e0165828. doi: 10.1371/journal.pone.0165828 (PMC5104331; doi:10.1371/journal.pone.0165828)

**Supplementary Methods.**

**Probability distribution**

Latent class analysis^(1)^ results in a set of parameters that specify a probability probability distribution of AF and the predictor variables combined. This probability distribution follows from the assumption of m latent classes, where within each latent class all variables are independent. This independence is called “local independence” and constitutes the basic principle of latent class models^(2)^. Hence, the latent class model is:

Pr(Y=y,X_1_=x_1_,…,X_p_=x_p_) = $\sum_{i=1}^{m} w_{i}p_{Y=y}^{i}\prod_{j=1}^{p} p_{X_{j}=x_{j}}^{i}$ (Eq. 1)

in which:

*m* is the number of latent classes.

*p* is the number of predictor variables.

*w_i_* is a parameter, which represents (in the latent class model) the probability that an arbitrary person belongs to latent class *i*.

$p_{Y=y}^{i}$ is a parameter, which represents the probability that the *Y* variable (i.e. AF in this application) has value *y* (i.e. 0 or 1).

$p_{X_{j}=x_{j}}^{i}$ is a parameter, which represents the probability that the *X_j_* variable (i.e. the *j*-th predictor variable in this application) has value *x_j_*.

The above equation is also implied by the equations in Henry’s and Lazarsfeld’s 1968 work^(3)^. From this multivariate probability distribution, the conditional probability of AF given the predictor variables *X_j_* can subsequently be derived, according to the definition of conditional probability:

Pr(*Y*=1|*X*_1_=*x*_1_,…,*X_p_*=*x_p_*)= Pr(*Y*=1,*X*_1_=*x_1_*,…,*X_p_*=*x_p_*)/ Pr(*X*_1_=*x*_1_,…,*X_p_*=*x_p_*) (Eq. 2)

Where Pr(*X*_1_=*x*_1_,…,*X_p_*=*x_p_*) is the marginal probability defined by : Pr(*X*_1_=*x*_1_,…,*X_p_*=*x_p_*) = Pr(*Y*=0,*X*_1_=*x*_1_,…,*X_p_*=*x_p_*) + Pr(*Y*=1,*X*_1_=*x*_1_,…,*X_p_*=*x_p_*).

Hence, latent class analysis can be used to predict the probability of AF conditional on the predictor variables and this is the basis of the risk predictions of AF in the latent class model.

**Maximum posterior probability**

Pr(*Y*=*y*,*X*_1_=*x*_1_,…,*X_p_*=*x_p_*) is the marginal distribution of a refined probability distribution

Pr(*Y*=*y*,*X*_1_=*x*_1_,…,*X_p_*=*x_p_*, *I*=*i* ) = $w_{i}p_{Y=y}^{i}\prod_{j=1}^{p} p_{X_{j}=x_{j}}^{i}$ (Eq. 3)

The probability of *I*=*i* conditional on AF and the predictors is:

Pr(*I*=*i* |*Y*=*y*, *X*_1_=*x*_1_,…,*X_p_*=*x_p_*)= Pr(*Y*=*y*,*X*_1_=*x*_1_,…,*X_p_*=*x_p_*, *I*=*i*)/ Pr(*Y*=*y*, *X*_1_=*x*_1_,…,*X_p_*=*x_p_*)

This is the “posterior probability” (because an alternative derivation is based on Bayes’ theorem and the calculation of a posterior probability).

If the parameters of a latent class model are given, it is possible to calculate for each person a posterior probability for each class *i*. It is then possible to assign to each person the class for which his posterior probability is largest. This is classification using maximum posterior probability.

**Parameter fitting and log likelihood**

From Eq. 1 follows that there *m*-1 independent parameters *w*_1_ to *w_m_*_-1_ (*w_m_* can be calculated from *w*_1_ to *w_m_*_-1_, because the sum of *w*_1_ to *w_m_* must be one), *m* independent parameters *p^i^_Y_*_=1_ for *i*= 1 to m (*p^i^_Y_*_=0_ = 1- *p^i^_Y_*_=1_), and for each *i* from 1 to m the (*L_j_*-1) parameters *p^i^_Xj_*_=_*_xj_*, where *L_j_* is the number of levels of *X_j_* (for each predictor variable there are only (*L_j_*-1) independent parameters because the *p^i^_Xj_*_=_*_xj_* of one arbitrarily selectable level *x_j_* can be computed from the other *p^i^_Xj_*_=_*_xj_*, because the sum over all possible values *x_j_* of *p^i^_Xj_*_=_*_xj_* must be one.

The parameters of the model can be estimated (for example) by the maximum likelihood method, which is maximizing $\prod_{i=1}^{N} Pr(Y=y^{i},X_{1}^{i}=x_{1}^{i},\ldots,X_{p}^{i}=x_{p}^{i})$ for the *N* persons in the fitting population, where *y^i^* = the AF status of person *i* and *x*_1_*^i^* to *x_p_^i^* are the predictors 1 to *p* for person *i*.

The log likelihood (LogL) is:

LogL = log($\prod_{i=1}^{N} Pr(Y=y^{i},X_{1}^{i}=x_{1}^{i},\ldots,X_{p}^{i}=x_{p}^{i}))$

**Bayesian information criterion and Akaike information criterion**

Maximizing the LogL cannot be used to determine the number of classes m, because it would generally lead to an unrealistically large m. The number of parameters used by the model increases with increasing m. In view of this, the Akaike information criterion (AIC) aims to find an optimum between number of parameters used by the model and high LogL:

AIC = 2*q*-2LogL

Where *q* is the number of estimated parameters used by the model.

The Bayesian information criterion (BIC) also accounts for the number of observations (*N*):

BIC = *q*log(*N*) – 2LogL

Minimizing AIC and BIC leads to a balance between high likelihood and low number of parameters.

**Root Mean Squared Error**

If a latent class model’s parameters have been found by fitting them to a population (e.g. by maximum likelihood), the model can be used to classify each individual using maximum posterior probability. After that, it is possible to use this classification to derive “estimated values” of the *w_i_*’s and $p_{Y=y}^{i}$, and $p_{X_{j}=x_{j}}^{i}$. The estimated values of the *w_i_*’s are the number of individuals in class *i* divided by the total number of individuals. The estimated values of the parameters $p_{Y=y}^{i}$ the proportion of people with AF status *y* in class *i*. And similarly, the estimated values of the parameters $p_{X_{j}=x_{j}}^{i}$ the proportion of people with *X_j_*=*x_j_* in class *i*. For each parameter, the “error” is the difference between the parameter and its estimated value.

It is then possible to calculate the Root Mean Squared Error (RMSE) as the mean square of these errors over all parameters of the model (i.e. all *w_i_*’s, and $p_{Y=y}^{i}$’s, and $p_{X_{j}=x_{j}}^{i}$’s).

The RMSE can be interpreted as a standard deviation associated with each model parameter and is therefore highly informative.

**Madansky**

The Madansky measure^(4)^ also starts with the maximum posterior probability classification. The Madansky measure aims to measure deviations from the local independence in this classification.

First, the estimated values of the $p_{Y=y}^{i}$’s, and $p_{X_{j}=x_{j}}^{i}$’s of the model are derived as was done for the RMSE.

Next, for each response pattern (i.e. a fixed set of values of *y* and *x*_1_ to *x_p_*) that occurs at least once in the population, its probability in each latent class is estimated using the latent parameters’ estimated values, so if the symbols *q* instead of *p* and *Q* instead of *P* are used to denote the estimated probability and if the response pattern *s* is (*y*,*x*_1_,…,*x_p_*), then the estimated probability of *s* in class *i* is:

*Q^i^*(*s*) = $q_{Y=y}^{i}\prod_{j=1}^{p} q_{X_{j}=x_{j}}^{i}$

The predicted number of people with this response pattern *s* in class *i* is then *Q^i^*(*s*) times the number of people in class *i*, denoted as *n_i_*.

The Madansky measure is now the sum over all squared differences between predicted numbers of response pattern and actual number of response pattern, weighted by the square of the class size *n_i_* divided by the estimated probability of *s* in the population.

**References**

1. P.F. Lazarsfeld & N.W. Henry (1968) Latent structure analysis. HOUGHTON MIFFLIN COMPANY, BOSTON. 294 pp.

2. J. Rost, R. Langeheine (Eds.) (1997) Applications of latent trait and latent class models in the social sciences. Waxmann Münster. 422 pp. Page 28.

3. P.F. Lazarsfeld & N.W. Henry (1968) Latent structure analysis. HOUGHTON MIFFLIN COMPANY, BOSTON. 294 pp. Page 47.

4. P.F. Lazarsfeld & N.W. Henry (1968) Latent structure analysis. HOUGHTON MIFFLIN COMPANY, BOSTON. 294 pp. Page 121.

**Supplementary Table A. PREVEND: Characteristics in the groups when each case is assigned to a group based on highest posterior probability of the latent class clustering analysis based on cardiovascular risk factors and diseases, including incident AF (primary analysis).**

| **Class** | **1 (n=1517)** | **2 (n=1482)** | **3 (n=1467)** | **4 (n=1228)** | **5 (n=1148)** | **6 (n=1082)** | **7 (n=341)** | **P-value** |
| --- | --- | --- | --- | --- | --- | --- | --- | --- |
| Age (years) | 36±5 | 40±9 | 60±8 | 45±7 | 50±7 | 62±8 | 65±8 | <0.001 |
| Age | |  |  |  |  |  |  |  |
| ≤35 years | 765(50.4%) | 602(40.6%) | 0(0.0%) | 86(7.0%) | 0(0.0%) | 5(0.5%) | 0(0.0%) |  |
| 36-43 years | 623(41.1%) | 499(33.7%) | 5(0.3%) | 413(33.6%) | 166(14.5%) | 3(0.3%) | 8(2.3%) |  |
| 44-50 years | 127(8.4%) | 226(15.2%) | 194(13.2%) | 489(39.8%) | 507(44.2%) | 55(5.1%) | 13(3.8%) |  |
| 51-61 years | 0(0.0%) | 84(5.7%) | 556(37.9%) | 240(19.5%) | 387(33.7%) | 403(37.2%) | 70(20.5%) |  |
| ≥62 years | 2(0.1%) | 71(4.8%) | 712(48.5%) | 0(0.0%) | 88(7.7%) | 616(56.9%) | 250(73.3%) | <0.001 |
| Antihypertensive therapy | 9(0.6%) | 6(0.4%) | 322(21.9%) | 56(4.6%) | 65(5.7%) | 379(35.0%) | 261(76.5%) | <0.001 |
| Men | 0(0.0%) | 1482(100.0%) | 1467(100.0%) | 842(68.6%) | 41(3.6%) | 0(0.0%) | 288(84.5%) | <0.001 |
| European ancestry | 1411(93.8%) | 1388(94.2%) | 1412(96.9%) | 1129(93.1%) | 1128(98.9%) | 1048(97.9%) | 328(97.6%) | <0.001 |
| Weight (kg) | 66 (61-74) | 79 (73-87) | 86 (80-95) | 82 (73-93) | 69 (63-76) | 77 (70-86) | 81 (75-89) | <0.001 |
| Length (cm) | 170 (165-174) | 182 (177-187) | 177 (173-182) | 176 (168-182) | 167 (163-172) | 164 (160-168) | 174 (169-180) | <0.001 |
| BMI |  |  |  |  |  |  |  |  |
| ≤22 kg/m^2^ | 704(47.1%) | 425(29.0%) | 38(2.6%) | 139(11.4%) | 275(24.3%) | 29(2.7%) | 24(7.1%) |  |
| 23-24 kg/m^2^ | 335(22.4%) | 421(28.7%) | 181(12.5%) | 208(17.0%) | 360(31.8%) | 83(7.8%) | 47(13.9%) |  |
| 25-26 kg/m^2^ | 194(13.0%) | 340(23.2%) | 351(24.2%) | 229(18.7%) | 247(21.8%) | 185(17.4%) | 90(26.6%) |  |
| 27-29 kg/m_2_ | 143(9.6%) | 200(13.6%) | 434(29.9%) | 309(25.3%) | 147(13.0%) | 297(27.9%) | 105(31.1%) |  |
| ≥30 kg/m^2^ | 120(8.0%) | 82(5.6%) | 448(30.9%) | 338(27.6%) | 104(9.2%) | 472(44.3%) | 72(21.3%) | <0.001 |
| Systolic BP (mmHg) | 112±11 | 122±11 | 144±20 | 135±15 | 118±15 | 143±22 | 140±24 | <0.001 |
| Diastolic BP (mmHg) | 66±6 | 69±6 | 83±8 | 81±7 | 69±7 | 77±8 | 76±9 | <0.001 |
| Diastolic BP | | |  |  |  |  |  |  |
| ≤68 mmHg | 1048(69.1%) | 672(45.4%) | 25(1.7%) | 0(0.0%) | 542(47.2%) | 152(14.0%) | 66(19.4%) |  |
| 69-76 mmHg | 415(27.4%) | 720(48.6%) | 285(19.5%) | 302(24.6%) | 477(41.6%) | 370(34.2%) | 133(39.0%) |  |
| ≥77 mmHg | 54(3.6%) | 89(6.0%) | 1155(78.8%) | 925(75.4%) | 129(11.2%) | 560(51.8%) | 142(41.6%) | <0.001 |
| Heart rate | |  |  |  |  |  |  |  |
| ≤63 bpm | 389(25.8%) | 737(50.2%) | 481(32.9%) | 59(4.8%) | 360(31.6%) | 212(19.6%) | 179(52.6%) |  |
| 64-72 bpm | 560(37.2%) | 524(35.7%) | 509(34.8%) | 414(33.8%) | 464(40.8%) | 377(34.9%) | 102(30.0%) |  |
| ≥73 bpm | 558(37.0%) | 208(14.2%) | 473(32.3%) | 752(61.4%) | 314(27.6%) | 490(45.4%) | 59(17.4%) | <0.001 |
| Alcohol use | 169(11.2%) | 45(3.0%) | 126(8.6%) | 216(17.7%) | 340(29.8%) | 143(13.3%) | 15(4.4%) | <0.001 |
| Heart failure | 0(0.0%) | 0(0.0%) | 0(0.0%) | 0(0.0%) | 0(0.0%) | 0(0.0%) | 18(5.3%) | <0.001 |
| Hypercholesterolemia | 35(2.3%) | 88(6.0%) | 327(22.4%) | 178(14.5%) | 105(9.2%) | 276(25.8%) | 226(67.1%) | <0.001 |
| Previous myocardial infarction | 1(0.1%) | 0(0.0%) | 17(1.2%) | 0(0.0%) | 3(0.3%) | 6(0.6%) | 224(66.5%) | <0.001 |
| Peripheral artery disease | 18(1.3%) | 18(1.3%) | 85(6.1%) | 16(1.4%) | 13(1.2%) | 69(6.8%) | 72(22.6%) | <0.001 |
| Diabetes mellitus | 2(0.1%) | 0(0.0%) | 112(7.8%) | 39(3.2%) | 0(0.0%) | 99(9.4%) | 58(17.4%) | <0.001 |
| Previous stroke | 2(0.1%) | 6(0.4%) | 29(2.0%) | 0(0.0%) | 12(1.1%) | 13(1.2%) | 19(5.7%) | <0.001 |
| PR interval duration | |  |  |  |  |  |  |  |
| ≤149 ms | 868(58.5%) | 428(29.7%) | 179(12.5%) | 432(36.0%) | 483(43.1%) | 247(23.5%) | 42(12.8%) |  |
| 150-166 ms | 362(24.4%) | 482(33.4%) | 370(25.8%) | 460(38.3%) | 357(31.9%) | 342(32.5%) | 74(22.5%) |  |
| ≥167 ms | 254(17.1%) | 532(36.9%) | 886(61.7%) | 308(25.7%) | 280(25.0%) | 464(44.1%) | 213(64.7%) | <0.001 |
| Serum creatinine (umol/l) | 73 (67-78) | 87 (81-94) | 94 (86-103) | 82 (71-92) | 78 (71-84) | 79 (71-86) | 95 (84-107) | <0.001 |
| Smoking | 798(52.9%) | 683(46.2%) | 450(30.9%) | 791(64.7%) | 531(46.6%) | 257(24.0%) | 160(47.6%) | <0.001 |
| Glomerular filtration rate (ml/min) | 83.8) (76.7-91.5) | 90.3 (82.9-98.1) | 75.5 (68.6-84.1) | 86.6 (79.5-95.1) | 73.4 (67.7-79.8) | 68.8 (61.8-77.7) | 71.6 (62.3-80.7) | <0.001 |
| Glomerular filtration rate | | |  |  |  |  |  |  |
| ≤74 ml/min | 271(18.0%) | 80(5.4%) | 682(46.7%) | 122(10.0%) | 634(55.5%) | 743(69.4%) | 202(59.6%) |  |
| 75-86 ml/min | 607(40.3%) | 419(28.5%) | 507(34.7%) | 461(37.9%) | 439(38.4%) | 211(19.7%) | 87(25.7%) |  |
| ≥87 ml/min | 627(41.7%) | 973(66.1%) | 272(18.6%) | 633(52.1%) | 69(6.0%) | 117(10.9%) | 50(14.7%) | <0.001 |
| Urinary albumin excretion ≥ 10 mg/L | 1042(68.7%) | 1006(67.9%) | 1168(79.6%) | 930(75.7%) | 565(49.2%) | 763(70.5%) | 285(83.6%) | <0.001 |
| Incident AF | 0(0.0%) | 4(0.3%) | 110(7.5%) | 2(0.2%) | 15(1.3%) | 45(4.2%) | 74(21.7%) | <0.001 |

Data are expressed as numbers (%), mean±SD, or median (25^th^ - 75^th^ percentile). Abbreviation: AF = atrial fibrillation, BMI = body mass index, BP = blood pressure.

**Supplementary Table B. PREVEND: The latent probabilities of the latent class model.**

| **Class** | **1** | **2** | **3** | **4** | **5** | **6** | **7** |
| --- | --- | --- | --- | --- | --- | --- | --- |
| Latent class size | 18.4% | 17.5% | 16.4% | 16.1% | 14.6% | 12.0% | 5.0% |
| Age |  |  |  |  |  |  |  |
| ≤ 36 years | 49.8% | 40.4% | 0.0% | 9.0% | 0.0% | 0.8% | 0.1% |
| 36 – 44 years | 37.6% | 33.2% | 1.2% | 31.7% | 20.6% | 1.2% | 2.3% |
| 44 – 51 years | 11.6% | 15.7% | 15.3% | 35.6% | 37.5% | 7.5% | 4.8% |
| 51 – 62 years | 0.6% | 5.7% | 36.4% | 22.8% | 32.2% | 37.8% | 20.6% |
| ≥ 62 years | 0.5% | 5.1% | 47.1% | 0.8% | 9.6% | 52.6% | 72.2% |
| Male | 1.4% | 99.9% | 100.0% | 68.2% | 8.7% | 0.0% | 83.2% |
| European ancestry | 94.2% | 94.9% | 96.9% | 93.9% | 98.5% | 98.1% | 97.3% |
| BMI |  |  |  |  |  |  |  |
| 16 – 22 kg/m^2^ | 46.1% | 27.8% | 2.7% | 12.0% | 24.1% | 3.3% | 6.5% |
| 22 – 24 kg/m^2^ | 23.0% | 28.4% | 13.5% | 17.3% | 29.6% | 9.4% | 13.5% |
| 24 – 26 kg/m^2^ | 13.2% | 23.6% | 23.7% | 19.5% | 21.2% | 18.3% | 24.7% |
| 26.6 – 29 kg/m^2^ | 9.5% | 13.9% | 29.9% | 25.0% | 13.9% | 26.5% | 31.3% |
| 29.2 – 59 kg/m^2^ | 8.2% | 6.2% | 30.1% | 26.2% | 11.2% | 42.4% | 24.1% |
| Diastolic blood pressure | |  |  |  |  |  |  |
| 47 – 69 mmHg | 69.2% | 44.7% | 2.1% | 0.0% | 46.1% | 16.7% | 16.4% |
| 69.0 – 77 mmHg | 26.8% | 46.7% | 19.2% | 31.2% | 39.3% | 33.9% | 35.5% |
| 77 – 121 mmHg | 3.9% | 8.6% | 78.6% | 68.8% | 14.6% | 49.4% | 48.1% |
| Heart rate |  |  |  |  |  |  |  |
| 30 – 64 bpm | 26.3% | 49.0% | 31.3% | 9.2% | 30.3% | 19.5% | 50.2% |
| 64 – 73 bpm | 37.5% | 35.9% | 35.0% | 34.4% | 40.3% | 35.2% | 31.0% |
| 73 – 115 bpm | 36.2% | 15.1% | 33.7% | 56.4% | 29.4% | 45.3% | 18.8% |
| Antihypertensive therapy | 0.8% | 0.5% | 21.7% | 4.8% | 5.7% | 33.2% | 73.3% |
| Previous myocardial infarction | 0.1% | 0.0% | 1.7% | 0.0% | 0.2% | 0.7% | 49.6% |
| Heart failure | 0.0% | 0.0% | 0.0% | 0.0% | 0.0% | 0.0% | 4.2% |
| Diabetes | 0.2% | 0.1% | 7.2% | 3.2% | 0.0% | 9.2% | 14.9% |
| Previous stroke | 0.2% | 0.4% | 1.9% | 0.0% | 0.9% | 1.1% | 5.0% |
| Peripheral artery disease | 1.2% | 1.4% | 5.6% | 1.4% | 1.4% | 6.4% | 18.4% |
| Smoking | 53.2% | 47.1% | 31.8% | 60.4% | 47.8% | 25.5% | 43.2% |
| Alcohol use | 12.5% | 3.5% | 8.9% | 16.9% | 26.0% | 14.1% | 4.7% |
| Hypercholesterolemia | 2.8% | 5.9% | 22.1% | 14.0% | 8.9% | 25.3% | 60.1% |
| PR interval duration | |  |  |  |  |  |  |
| 93 – 150 ms | 57.7% | 29.1% | 13.5% | 35.7% | 42.2% | 24.2% | 12.0% |
| 150 – 167 ms | 24.6% | 33.4% | 26.7% | 36.2% | 31.1% | 32.8% | 23.3% |
| 167 – 290 ms | 17.7% | 37.5% | 59.8% | 28.0% | 26.7% | 43.0% | 64.7% |
| eGFR-creatinin-based |  |  |  |  |  |  |  |
| 4.51 - 74.43 ml/min | 19.1% | 6.0% | 44.4% | 14.0% | 52.5% | 67.8% | 59.0% |
| 74.43 - 86.09 ml/min | 40.4% | 27.9% | 35.1% | 37.6% | 38.2% | 20.5% | 25.8% |
| 86.09 - 271.75 ml/min | 40.6% | 66.1% | 20.5% | 48.4% | 9.2% | 11.7% | 15.2% |
| UAC ≥ 10 mg/L | 68.6% | 68.2% | 80.3% | 75.5% | 52.9% | 70.1% | 83.5% |
| AF | 0.0% | 0.4% | 6.8% | 0.3% | 1.0% | 3.7% | 18.9% |

Abbreviations: AF = atrial fibrillation; BMI = body mass index; UAC = urinary albumin excretion, eGFR = estimated glomerular filtration rate.

**Supplementary Table C. Multivariable-adjusted Cox proportional hazards regression coefficients for 10-year risk of AF.**

|  | **PREVEND** | **Framingham** |
| --- | --- | --- |
| Age | 0.093 (0.008) | 0.076 (0.011) |
| European ancestry | -0.915 (0.377) | - |
| Height | 0.028 (0.012) | 0.002 (0.013) |
| Weight | 0.023 (0.006) | 0.011 (0.005) |
| Systolic blood pressure | 0.012 (0.005) | 0.013 (0.005) |
| Diastolic blood pressure | -0.022 (0.011) | -0.024 (0.009) |
| Smoking | 0.126 (0154) | 0.517 (0.196) |
| Antihypertensive treatment | 0.417 (0.162) | 0.585 (0.160) |
| Diabetes | 0.010 (0.250) | 0.306 (0.204) |
| Heart failure | 1.201 (0.471) | 0.901 (0.568) |
| Myocardial infarction | 0.667 (0.219) | 0.622 (0.303) |
| Urinary albumin excretion ≥ 10 mg/l | 0.018 (0.177) | - |
| Men | 0.183 (0.227) | 0.377 (0.227) |
| Mean linear predictor | 10.577 | - |

Data are expressed as bèta (SD).

**Supplementary Figure A. Graphical representation of the latent class model with distal outcome (see also Lanza et al, 2013).** C refers to the latent class variable. The class-defining variables of C are age (shown in the figure), men (shown in the figure), European ancestry, body mass index, diastolic blood pressure, heart rate, antihypertensive treatment, Previous myocardial infarction, heart failure, diabetes, previous stroke, peripheral artery disease, smoking, alcohol use, hypercholesterolemia, ECG PR interval duration, eGFR-creatinine-based <60, and UAC ≥ 10 mg/L (shown in the figure). The outcome is incident AF.


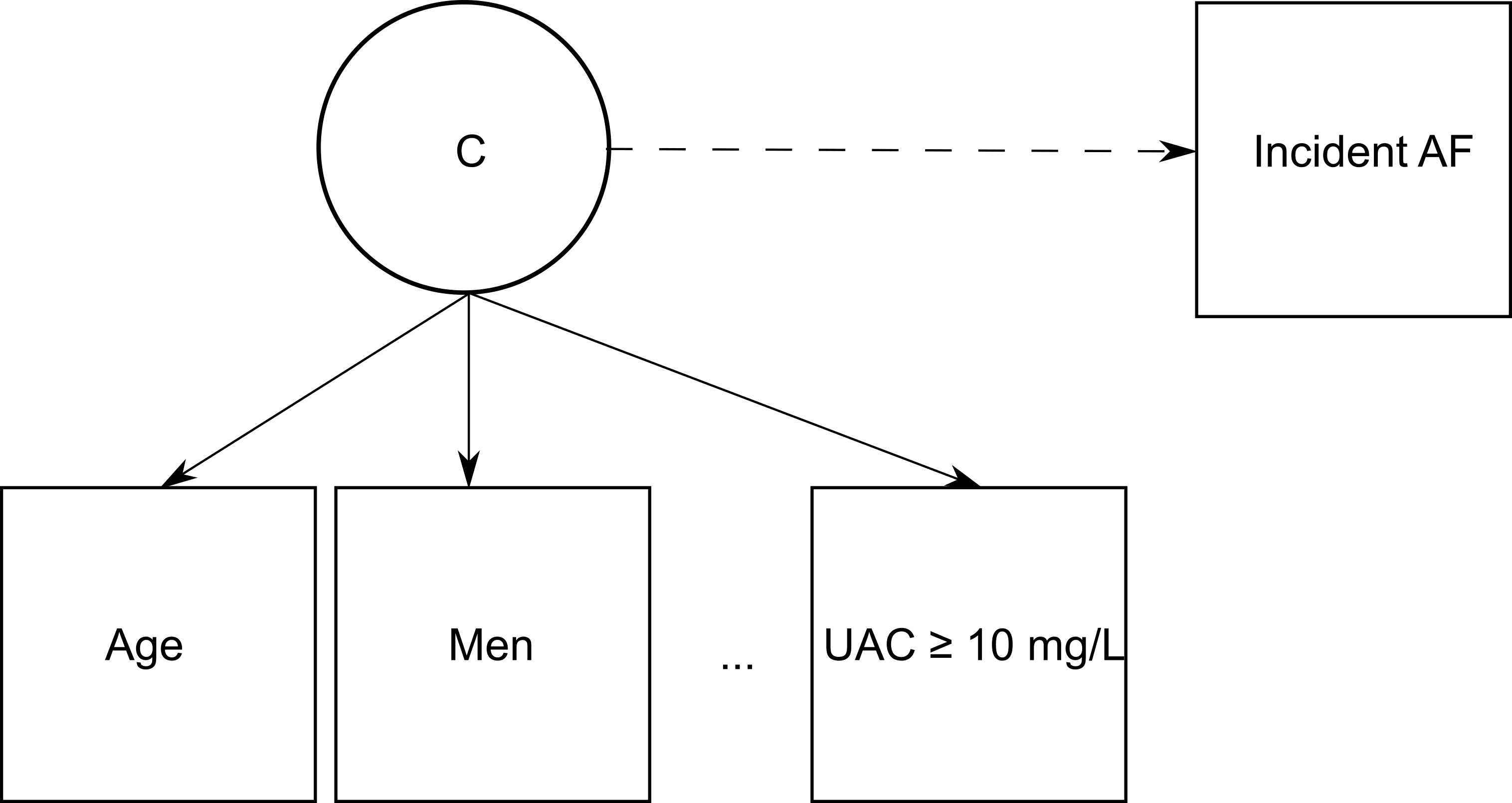


**Supplementary Figure B. Underlying cumulative hazard function of the traditional risk factor-based model.** The PREVEND population was used to estimate the underlying cumulative hazard function of the traditional risk factor-based model. The solid line is the underlying cumulative hazard function of the traditional risk factor-based model, the dashed lines represent the 95% confidence interval.


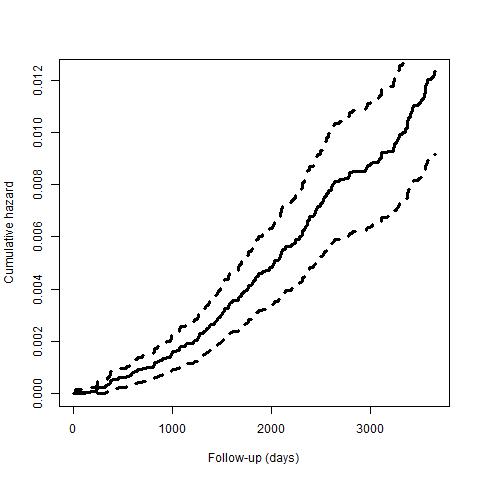

Supplement: S1 File — Table A. PREVEND: Characteristics in the groups when each case is assigned to a group based on highest posterior probability of the latent class clustering analysis based on cardiovascular risk factors and diseases, including incident AF (primary analysis). Table B. PREVEND: The latent probabilities of the latent class model. Table C. Multivariable-adjusted Cox proportional hazards regression coefficients for 10-year risk of AF. Fig A: Graphical representation of the latent class model with distal outcome. C refers to the latent class variable. The class-defining variables of C are age (shown in the Fig), men (shown in the Fig), European ancestry, body mass index, diastolic blood pressure, heart rate, antihypertensive treatment, Previous myocardial infarction, heart failure, diabetes, previous stroke, peripheral artery disease, smoking, alcohol use, hypercholesterolemia, ECG PR interval duration, eGFR-creatinine-based <60, and UAC ≥ 10 mg/L (shown in the Fig). The outcome is incident AF. Fig B: Underlying cumulative hazard function of the traditional risk factor-based model. The PREVEND population was used to estimate the underlying cumulative hazard function of the traditional risk factor-based model. The solid line is the underlying cumulative hazard function of the traditional risk factor-based model, the dashed lines represent the 95% confidence interval. (DOCX) [file pone.0165828.s001.docx]
